# Evaluating the prognostic contributions of TNM classifications and building novel staging schemes for middle ear squamous cell carcinoma

**DOI:** 10.1002/cam4.4306

**Published:** 2021-09-24

**Authors:** Ke Qiu, Wendu Pang, Jianqing Qiu, Junhong Li, Danni Cheng, Yufang Rao, Yijun Dong, Minzi Mao, Qiurui Liu, Xiaosong Mu, Wei Zhang, Wei Xu, Jianjun Ren, Yu Zhao

**Affiliations:** ^1^ Department of Oto‐Rhino‐Laryngology West China Hospital Sichuan University Chengdu China; ^2^ West China Biomedical Big Data Center West China Hospital Sichuan University Chengdu China; ^3^ Medical Big Data Center Sichuan University Chengdu China; ^4^ Department of Oto‐Rhino‐Laryngology Langzhong People’s Hospital Langzhong China; ^5^ Department of Biostatistics Princess Margaret Cancer Centre and Dalla Lana School of Public Health Toronto Ontario Canada

**Keywords:** middle ear squamous cell carcinoma, prognoses, SEER, staging scheme, Stell’s classification

## Abstract

**Background:**

A universally acknowledged cancer staging system considering all aspects of the T‐, N‐, and M‐classifications for middle ear squamous cell carcinoma (MESCC) remains absent, limiting the clinical management of MESCC patients.

**Materials and Methods:**

A total of 214 MESCC patients were extracted from the SEER (the Surveillance, Epidemiology, and End Results) database between 1973 and 2016. The relationships between patient’s characteristics and prognoses were analyzed by Kaplan–Meier and Cox proportional hazards regression models. Novel staging schemes for MESCC were designed by adjusted hazard ratio (AHR) modeling method according to the combinations of Stell’s T‐classification and the eighth AJCC N‐ and M‐classifications, of which performances were evaluated based on five criteria: hazard consistency, hazard discrimination, explained variation, likelihood difference, and balance.

**Results:**

T‐classification was the most significant prognostic factor for MESCC patients in multivariable analysis (*p* = 0.021). The N‐ and M‐classifications also had obvious prognostic effect but were not statistically significant by multivariate analysis due to the limited metastasis events. Three novel staging schemes (AHR‐Ⅰ–Ⅲ models, different combination of T‐ and N‐classifications) and ST (solely derived from Stell’s T‐classification) were developed, among which the AHR‐Ⅰ staging scheme performed best.

**Conclusions:**

Tumor extension, quantified by Stell’s T‐classification, is the most significant prognostic factor for MESCC patients. However, our AHR‐Ⅰ staging scheme, a comprehensive staging scheme that integrating T‐, N‐, and M‐classifications, might be an optimal option for clinical practitioners to predict MESCC patients’ prognosis and make proper clinical decisions.

## Graphical abstract

All of Stell’s T‐classification, N‐ and M‐classifications of the eighth AJCC staging system for head and neck cancer showed obvious prognostic effects in middle ear squamous cell carcinoma (MESCC) patients. Stell’s T‐classification is the most significant as well as an independent prognostic factor for MESCC patients. Our newly developed AHR‐Ⅰ staging scheme displayed better performance than the Stell’s T‐classification in almost all existing criterion, which stresses the importance of a comprehensive staging scheme integrating T‐, N‐, and M‐classifications for MESCC and provides a new perspective for clinical practitioner who solely rely on tumor extension for making clinical decisions.

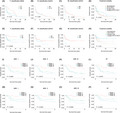

## 1 INTRODUCTION

Temporal bone carcinoma (TBC) is an uncommon aggressive malignancy that accounts for less than 0.2% of tumors originated from head and neck region.^1^ It is estimated that the majority of TBC arise from external auditory canal (EAC), while middle ear carcinoma (MEC) only represents a small subset of TBC, of which squamous cell carcinoma (SCC) is the predominated histologic type and remained to be explored.^2^, ^3^

Due to the rarity of middle ear squamous cell carcinoma (MESCC) and the fact that its metastasis progresses quite slow, it is difficult to observe lymph nodes or distant metastasis in MESCC cohorts without sufficient follow‐up time.^4^, ^5^, ^6^ As a result, there is few uniform cancer staging standard for MESCC, and clinical practitioners usually have to solely rely on the tumor status (T‐classification) to guide the selection of management strategies and predict patients’ outcomes. Since a reliable cancer staging system is able to distinguish patients with different prognoses and guide the selection of management strategies before treatment starts,^7^, ^8^, ^9^ researchers focusing on the staging system of MESCC have never ceased their steps.

Currently, a few staging systems have been proposed for MESCC, among which the most recognized are the Pittsburgh staging system and Stell’s T‐classification system. However, due to the small sample sizes (<100 patients) and limited clinical variables taken into account, neither of them was endorsed by the American Joint Committee on Cancer (AJCC) or the International Union Against Cancer (UICC) TNM staging systems.^10^, ^11^, ^12^ Besides, most of those staging classification studies mixed external auditory canal carcinoma (EACC) and MEC together, the tumor nature of which was largely different.^4^, ^5^, ^13^, ^14^ Specifically, the Pittsburgh staging system, the most commonly used staging system for TBC, was established on the basis of a retrospective study that only included EACC data, therefore, their application value for MESCC remained elusive.^5^, ^12^, ^13^, ^14^ Nevertheless, the T‐classification proposed by Stell et al. was proved to be effective in distinguishing MESCC patients with different prognoses, however, it did not consider the contributions of lymph nodes and distant metastasis.^6^, ^10^, ^15^ To what extent the N‐ and M‐classifications would affect MESCC patients’ survival outcomes remains unknown.

The absence of a universally accepted staging system for MESCC may impede rational comparison of clinical studies and corresponding reported treatment efficacy. In order to assist guiding the clinical management of MESCC patients and predicting their prognoses comprehensively, we developed several novel staging systems for MESCC patients by integrating lymph nodes and distant metastasis (N‐ and M‐classifications determined by eighth AJCC staging system) status into Stell’s T‐classification system, and validate them by a refined evaluation methodology proposed by Xu et al, which had been successfully applied to develop the staging schemes for oropharyngeal carcinoma.^16^, ^17^

## 2 MATERIALS AND METHODS

### 2.1 Eligibility criteria and data extraction

Data were extracted from the Surveillance, Epidemiology, and End Results (SEER) Program from 1973 to 2016 using SEER*‐Stat version 8.3.5 (National Cancer Institute). Participants were selected according to the primary site category of “middle ear,” with ICD‐O3 code to be C30.1. The exclusion criteria were as follows: (1) tumor was identified on the death certificate only; (2) pathological results were not SCC; and (3) survival was less than 1 month.

A total of 214 MESCC patients diagnosed between 1973 and 2016 were identified from SEER database. Demographic and tumor‐specific data including marital status, age at diagnosis, gender, ethnicity, histology, tumor grades, extent of disease, treatment modalities, and survival outcomes were extracted. Institutional review board (IRB) approval was waived because SEER is a de‐identified governmental database. Data were extracted and reported in accordance with the SEER database user agreement.

### 2.2 The T‐, N‐, and M‐classification strategies

The T‐classification for MESCC was generated according to the staging system devised by Stell et al^10^ (referred as T‐classification in the following text), the details of which are presented in Table S1 along with the information about how to convert extent of disease from SEER into Stell’s T‐classification. Meanwhile, the lymph nodes (N) and metastases (M) classifications were derived according to the eighth AJCC staging system^18^ (referred as N‐ and M‐classifications in the following text) as below: N0: No regional lymph node metastasis; N1–3: Presence of regional or distant lymph node metastasis (because the data from SEER lack the information of size, laterality, and accurate metastasis sites of lymph nodes, we had to merge the N1, N2, and N3 together as a group of N1–N3); M0: No distant metastasis; M1: Presence of distant metastasis.

### 2.3 Modeling and evaluating strategies for staging scheme

We developed these novel MESCC staging systems according to the adjusted hazard ratios (AHRs) acquired by multivariable Cox regression model, which calculated adjusted HRs for risk of death with various combinations of T‐ and N‐classifications among M0 patients. Meanwhile, we also considered minimum hazard difference, the ordinal order of T‐ and N‐classifications, and the sample size balance between stage subgroups. The performance of each staging scheme was evaluated with respect to survival according to five established criteria: hazard consistency, hazard discrimination, explained variation, likelihood difference, and balance.^16^, ^17^, ^19^

Briefly, hazard consistency demonstrates how well the stage groups represent subgroups. Hazard discrimination demonstrates the linear trend in log hazard ratio from the first stage group to the last stage group. Explained variation represents the proportion of the variation of censored survival times explained by a specific proportional hazard model. Likelihood difference demonstrates the improvement of fit of the model with stage grouping and clinical variables compared to the model that only contains clinical variables. Balance examines whether there was an equal number of patients in each group. The actual measure of each criterion was normalized and higher rank along with lower actual measure and score indicates better performance in each criterion except for likelihood difference and explained variation, of which higher actual measure indicates better performance. Finally, the five criteria‐based scores of each AHR stage were added to achieve an overall score and we ranked all of the AHR stages according to their overall scores, with the lowest score ranking first.

### 2.4 Statistical analysis

Descriptive statistics were provided with median and range for continuous factors and frequencies and percentages for categorical factors. Demographics and clinical characteristics were compared by the Kruskal–Wallis tests for continuous variables and Fisher's exact tests for categorical variables. The Kaplan–Meier (K–M) method was used to depict overall survival (OS) and cause‐specific survival (CSS), and Cox proportional hazards model and the competing risk method were fitted to depict the association between baseline characteristics and prognoses (OS and CSS). Two‐tailed tests were used and *p* values <0.05 were considered as significant. Variables adjusted in the multivariable regression model were selected based on the results of univariable analysis.

## 3 RESULTS

### 3.1 Baseline characteristics

As shown in Table 1, among the included 214 MESCC patients (58% male), of which nearly 3/4 were aged over 60 years old. The majority of MESCC patients progressed to T3 (65%), while only 15% patients presented with lymph node metastasis and 5% patients had distant metastasis. Besides, approximately 1/3 of those patients received adjuvant therapy only, followed by surgery plus postoperative adjuvant therapy (31%) and surgery only (24%).

**TABLE 1:** Baseline characteristics of MESCC patients (full sample size, *n* = 214)

Covariate	Number, %
Marital status	
Married	171 (87)
Unmarried	25 (13)
Missing	18
Race	
White	169 (79)
Black	14 (7)
Others	28 (13)
Missing	3
T‐classification	
T1	23 (15)
T2	30 (20)
T3	97 (65)
Missing	64
N‐classification	
N0	113 (85)
N1–3	20 (15)
Missing	81
M‐classification	
M0	146 (95)
M1	8 (5)
Missing	60
Age	
<60	59 (28)
60–69	59 (28)
>=70	96 (45)
Gender	
Female	89 (42)
Male	125 (58)
Grade	
Grade 1	55 (34)
Grade 2	73 (45)
Grade 3	31 (19)
Grade 4	2 (1)
Missing	53
Treatment	
No Treatment	24 (11)
S only	52 (24)
RT/CT/RCT	71 (33)
S+RT/CT/RCT	67 (31)

Abbreviations: CRT, chemoradiation; CT, chemotherapy; MESCC, middle ear squamous cell carcinoma; RT, radiotherapy; S, surgery.

### 3.2 Univariable and multivariable Cox regression analyses for survival outcomes

Univariate analysis showed that gender, treatment modality, Stell's T‐classification, and M‐classification were significant prognostic factors for both OS and CSS of MESCC patients (Table S2). While after adjustment for potential confounding factors, only Stell's T‐classification remained as an independent prognostic factor for OS of MESCC patients, with worse prognosis being indicated by higher stage (Table 2, T2 vs. T1: HR: 1.90, 95%CI: 0.78–4.70, *p* = 0.15; T3 vs. T1: HR: 3.00, 95%CI: 1.34–6.72, *p* = 0.0077).

**TABLE 2:** Multivariable Cox model of OS and CSS in MESCC patients

Covariate	OS			CSS		
	HR (95%CI)	*p* value	Global *p* value	HR (95%CI)	*p* value	Global *p* value
Age						
<60	Reference		0.075	Reference		0.42
60–69	0.79 (0.43, 1.45)	0.44		1.11 (0.54, 2.28)	0.78	
>=70	1.47 (0.87, 2.47)	0.15		0.66 (0.29, 1.48)	0.31	
Treatment						
No treatment	Reference		0.64	Reference		0.99
RT/CT/CRT	0.61 (0.24, 1.52)	0.29		1.02 (0.23, 4.62)	0.98	
S only	0.65 (0.24, 1.74)	0.39		1.04 (0.20, 5.52)	0.96	
S+RT/CT/CRT	0.79 (0.32, 1.91)	0.60		1.13 (0.25, 5.02)	0.88	
Gender						
Female	Reference		0.56	Reference		0.14
Male	0.86 (0.53, 1.40)	0.56		0.61 (0.32, 1.17)	0.14	
T‐classification						
T1	Reference		0.021*	Reference		0.075
T2	1.90 (0.78, 4.70)	0.15		5.65 (0.66, 48.22)	0.11	
T3	3.00 (1.34, 6.72)	0.0077*		9.26 (1.19, 72.3)	0.034*	
N‐classification						
N0	Reference		0.33	Reference		0.60
N1–3	1.42 (0.7, 2.88)	0.33		1.25 (0.54, 2.91)	0.60	
M‐classification						
M0	Reference		0.35	Reference		NA
M1	3.01 (0.3, 30.38)	0.35		NA	NA	

Abbreviations: CRT, chemoradiation; CSS, cause‐specific survival; CT, chemotherapy; MESCC, middle ear squamous cell carcinoma; OS, overall survival; RT, radiotherapy; S, surgery.

* *p* < 0.05.

**FIGURE 1:**
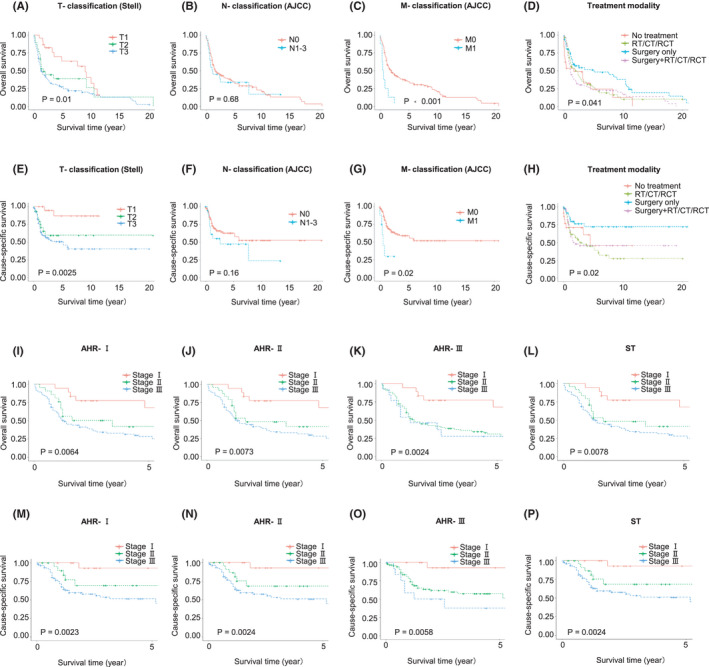
K–M curves in MESCC patients by different groups and different staging schemes. (A) K–M curves of overall survival (OS) in MESCC patients grouped by T‐classification, (B) K–M curves of OS in MESCC patients grouped by N‐classification, (C) K–M curves of OS in MESCC patients grouped by M‐classification, (D) K–M curves of OS in MESCC patients grouped by treatment modality, (E) K–M curves of cause‐specific survival (CSS) in MESCC patients grouped by T‐classification, (F) K–M curves of CSS in MESCC patients grouped by N‐classification, (G) K–M curves of CSS in MESCC patients grouped by M‐classification, (H) K–M curves of CSS in MESCC patients grouped by treatment modality, (I) K–M curves of overall survival (OS) in MESCC patients grouped by AHR‐Ⅰ stage, (J) K–M curves of OS in MESCC patients grouped by AHR‐Ⅱ stage, (K) K–M curves of OS in MESCC patients grouped by AHR‐Ⅲ stage, (L) K–M curves of OS in MESCC patients grouped by ST stage, (M) K–M curves of cause‐specific survival (CSS) in MESCC patients grouped by AHR‐Ⅰ stage, (N) K–M curves of CSS in MESCC patients grouped by AHR‐Ⅱ stage, (O) K–M curves of CSS in MESCC patients grouped by AHR‐Ⅲ stage, and (P) K–M curves of CSS in MESCC patients grouped by ST stage

As shown in Table S3, the prognoses worsened with growing age and female patients showed significant worse prognosis than male patients (5‐year OS: 18% vs. 41%, 5‐year CSS: 42% vs. 59%). Besides, patients treated with surgery alone had far better prognoses than patients treated with adjuvant therapy alone (5‐year OS: 51% vs. 27%, 5‐year CSS: 72% vs. 45%), which might be due to the more advanced stages of patients treated with adjuvant therapy alone (Figure 1D and 1H, Table S3).

Meanwhile, as expected, the survival curves of MESCC patients based on the T‐, N‐, and M‐classifications gradually declined as the stages increased (Figure 1A–C and Figure 1E–G, Table S3). Specifically, those T3 patients had obvious poorest prognoses (5‐year OS: 27%, 5‐year CSS:51%) than patients who were T2 (5‐year OS: 62%, 5‐year CSS:59%) and T1 (5‐year OS: 63%, 5‐year CSS: 87%); N0 patients had far better prognoses than N1–3 patients (5‐year CSS: 63% vs. 47%); and M1 patients showed exceedingly worse prognoses than M0 patients (5‐year OS: 0% vs. 51%, 5‐year CSS 30% vs. 59%). T‐classification was consistently of significance in both univariate analysis and multivariate analysis, while N‐ and M‐classifications showed obvious risk (HR>1) but not of significance (*p* < 0.05), which might largely be due to the limited sample size (only 20 patients had N1–3 and 8 patients had M1).

### 3.3 Development of novel staging schemes

**FIGURE 2:**
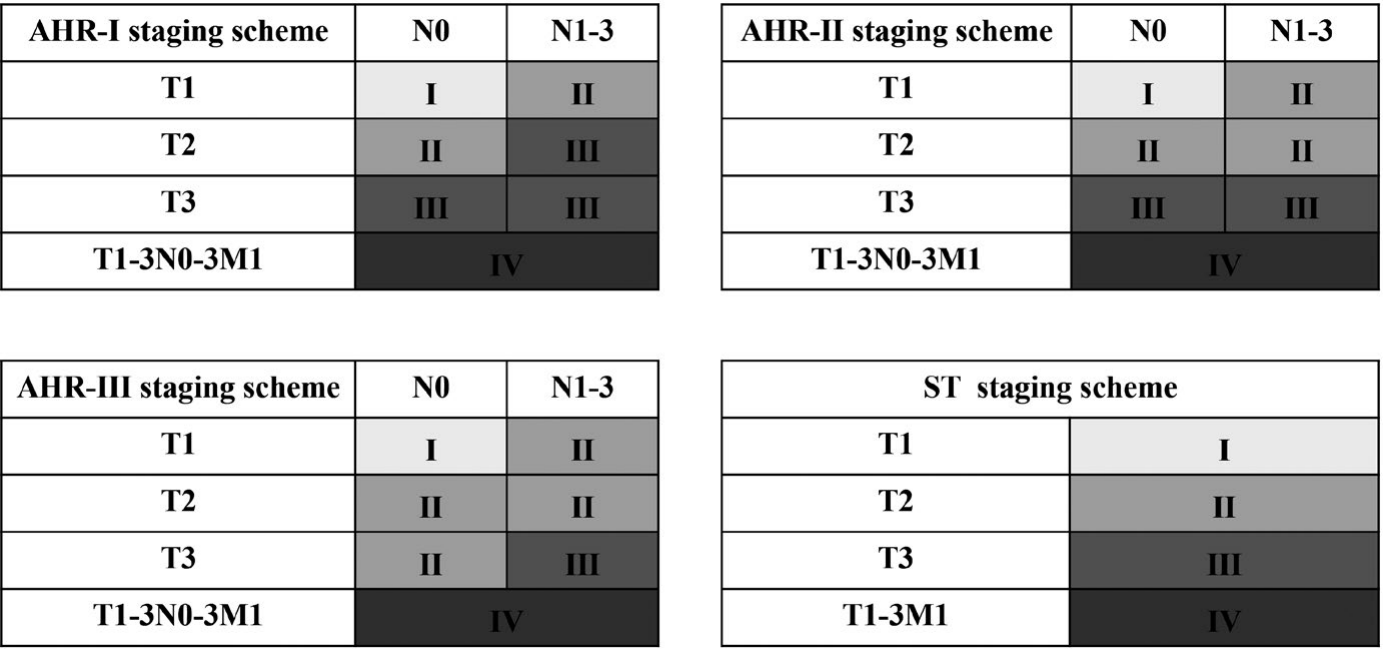
Schematic presentation of four staging schemes for MESCC patients. (Same staging schemes were generated based on both of overall survival and cause‐specific survival). Notes: Stages Ⅰ–Ⅲ were generated based on different combinations of T‐ and N‐classifications in M0 patients, while stage Ⅳ was generated solely in M1 patients regardless of T‐ and N‐classifications

Three AHR staging schemes (AHR‐Ⅰ–AHR‐Ⅲ) were generated according to the similarity of HRs for OS and CSS, respectively, which were adjusted for significant prognostic factors in corresponding univariate analyses (details of AHR and distribution of MESCC patients based on different combinations are shown in Table S4,S5). Additionally, considering the importance of Stell’s T‐classification in current clinical practice, we generated a clinical staging scheme (ST) solely dependent on Stell’s T‐classification for better comparison with other AHR staging schemes. And these four staging schemes (AHR‐Ⅰ–Ⅲ and ST) as well as their corresponding K–M curves of OS and CSS are presented in Figure 1I–P and Figure 2, respectively. As expected, the survival curves of both OS and CSS in MEC patients based on these four staging schemes gradually declined as stage progressed (shown in Table S3). In addition, M1 patients showed significantly worse prognoses than stage Ⅲ patients based on all of the four staging schemes (5‐year OS: 0% vs. 28% for AHR‐Ⅰ, 28% for AHR‐Ⅱ, 28% for AHR‐Ⅲ and 28% for ST; 5‐year CSS: 30% vs. 51% for AHR‐Ⅰ, 51% for AHR‐Ⅱ, 38% for AHR‐Ⅲ and 51% for ST), thus a separate stage IV was directly assigned to M1 patients regardless of T‐ and N‐classifications (Table S3).

### 3.4 Performance evaluation of novel staging schemes under five criteria

Four staging schemes based on OS were evaluated according to their performance under five different criteria (Table 3): (1) Hazard consistency: The difference in model fitting statistic among these four staging schemes ranged from a low of 0.45 for the AHR‐Ⅰ, demonstrating the best hazard consistency, to a high of 1.18 for the AHR‐Ⅲ, demonstrating the worst consistency. (2) Hazard discrimination: The hazard discrimination measure varied from 0.03 for AHR‐Ⅰ to 1.02 for AHR‐Ⅲ (the worst scheme by this criterion). (3) Explained variation: After adjusted for important clinical confounders, AHR‐Ⅰ was the best scheme for predicting the hazard associated with MESCC, with 6.97% of the variance in survival explained. AHR‐Ⅲ scheme did worst, with 6.07% of the variance explained. (4) Likelihood difference: The likelihood difference measure ranged from 5.45 for AHR‐Ⅰ to 4.72 for AHR‐Ⅲ (the worst scheme). (5) Balance: AHR‐Ⅱ and ST did best in splitting the patient population into evenly sized groups, with a deviation score of 0.64. AHR‐Ⅲ did worst at 0.83. (6) Summary scores: Table 3 also presents the standardized, weighted scores for each criterion along with the summary score and rankings. A higher overall score indicated a worse scheme performed.

**TABLE 3:** Performance evaluation of staging schemes based on refined methodology

Evaluation criteria	ST		AHR‐Ⅰ		AHR‐Ⅱ		AHR‐Ⅲ	
	OS	CSS	OS	CSS	OS	CSS	OS	CSS
1. Hazard consistency	0.47	0.30	0.45	0.10	0.47	0.30	1.18	1.00
Score	0.03	0.23	0.00	0.00	0.03	0.23	1.00	1.00
Rank	2	2	1	1	2	2	4	4
2. Hazard discrimination	0.09	0.87	0.03	0.54	0.09	0.87	1.02	3.81
Score	0.06	0.10	0.00	0.00	0.06	0.10	1.00	1.00
Rank	2	2	1	1	2	2	4	4
3. Likelihood difference	5.43	5.03	5.45	5.24	5.43	5.03	4.72	4.33
Score	0.03	0.23	0.00	0.00	0.03	0.23	1.00	1.00
Rank	2	2	1	1	2	2	4	4
4. Outcome prediction (% variance explained)	6.62	10.31	6.97	10.70	6.62	10.31	6.07	8.83
Score	0.21	0.21	0.00	0.00	0.21	0.21	1.00	1.00
Rank	2	2	1	1	2	2	4	4
5. Balance	0.64	0.64	0.69	0.69	0.64	0.64	0.83	0.83
Score	1.00	0.00	0.00	0.27	1.00	0.00	0.00	0.00
Rank	1	1	3	3	1	1	4	4
Overall score	0.60	0.54	0.27	0.27	0.60	0.54	3.50	4.00
Overall rank	2	2	1	1	2	2	4	4

Note

ST: a staging scheme solely based on Stell's T‐classification; AHR‐Ⅰ–Ⅲ: novel staging schemes developed based on adjusted hazard ratio modeling method according to different combinations of T‐ and N‐classifications. The actual measures and standardized, weighted scores were presented in the first and second row of each criteria, respectively. And higher rank along with lower actual measure and score indicates better performance in each criterion except for likelihood difference and explained variation, of which higher actual measure indicates better performance. The actual score of each criterion was normalized and the five criteria‐based scores of each AHR stage were added to achieve an overall score and we ranked all of the AHR stages according to their overall scores, with the lowest score ranking first.

Overall, the AHR‐Ⅰ (stage Ⅰ for T1N0M0, stage Ⅱ for T1N1‐3M0 and T2N0M0, stage Ⅲ for T2N1‐3M0, T3N0M0 and T3N1‐3M0, and stage IV for T1‐3N0‐3M1) performed best in all of the five criteria except for balance (third best performance) and therefore ranked first overall (overall score: 0.27). Whereas AHR‐Ⅲ performed worst in all of the criteria and ranked last with an overall score of 3.50. Meanwhile, performance evaluation of novel staging schemes based on CSS presented with similar results, of which AHR‐Ⅰ remained to rank first (overall score: 0.27), whereas AHR‐Ⅲ performed worst in all of the criteria and ranked last with an overall score of 4.00.

## 4 DISCUSSION

In this study, we comprehensively explored the prognostic effects of Stell’s T‐, eighth AJCC N‐ and M‐classifications as well as other clinical factors in affecting MESCC patients’ survival outcomes, and confirmed that Stell’s T‐classification was a strong and independent prognostic factor for the OS of MESCC patients. Due to the rarity of MEC, most of the previous studies investigated their prognostic factors in samples with mixed histological types and consistently revealed that SCC was associated with significantly worse prognosis, which also displayed highly distinguished biological characteristics.^2^, ^20^, ^21^, ^22^, ^23^ However, few studies systematically investigated the prognostic factors, survival outcomes, and staging schemes in MESCC patients alone. Only Feng et al. reported a cohort of 18 MESCC patients, in which Stell’s T‐classification was proved to show efficacies in treatment guidance and prognosis prediction,^24^ while further studies with larger sample sizes were still needed. This study included 214 MESCC patients with long‐term follow‐ups for analyses, and confirmed that the prognostic value of Stell’s T‐classification which had been previously described in MEC with mixed histological types, could also be generalized to pure MESCC.^4^, ^6^

Notably, in our study, the contribution of N‐ and M‐classifications was largely overwhelmed by the T‐classification, even though they also showed obvious prognostic effects but not of significance, which might be due to two reasons: first was the rarity of metastasis events. MESCC progressed quite slow, and metastasis was not commonly observed,^4^, ^5^, ^6^ among which only 13% patients had lymph node metastasis and 6% had distant metastasis in our report. Second, most of the patients who had lymph node metastasis (82% N1–3) or distant metastasis (100% M1) were at T3 classification, which might result in high overlapping rates and interaction between these three classifications. Thus, further larger MESCC cohort that observed enough metastasis events would be needed to explore the role of N‐ and M‐classifications.

On the other hand, due to the absence of a uniform staging system, clinicians have never ceased their steps to explore novel staging schemes and also investigate their scope of application. The Stell’s T‐classification was first proposed by Stell et al. in 1985.^10^ However, their staging system was generated by assessing a small amount of heterogeneous temporal bone squamous cell carcinoma (TBSCC, including MESCC and SCC of external auditory canal [EACSCC]) patients in one single institute, therefore, the effects of applying their staging system to other pure MESCC patients were relatively unstable.^10^ Similarly, another widely used staging system, the MPB Staging System, initially derived from EACSCC, was also confirmed to be a significant prognostic factor for TBSCC by numerous studies.^13^, ^14^, ^25^, ^26^, ^27^, ^28^, ^29^, ^30^, ^31^, ^32^ However, most of these studies only confirmed that the MPB T‐classification, rather than the overall stage, was a significant prognostic factor. Besides, most of these previous studies only included EACSCC or a mixed population of TBSCC, thus whether the MPB is proper for the staging of pure MESCC remain to be further clarified.

Therefore, we developed three novel staging schemes (AHR‐Ⅰ–Ⅲ) according to AHR modeling method by incorporating different Stell's T‐classification, the eighth AJCC N‐ and M‐classifications, and compared their efficacy with the ST stage (derived solely from Stell's T‐classification) by applying five refined criteria. As a result, the AHR‐Ⅰ stage performed best in all of the five criteria except for the balance (third best performance) and therefore ranked first overall, which stressed the importance of a comprehensive staging scheme integrating T‐, N‐, and M‐classifications for MESCC.

Additionally, we applied an existing refined methodology to incorporate important clinical factors into the new criteria and applied the parametric approach to evaluate staging schemes using the likelihood ratio statistic from Cox proportional hazards model. This method also improved the existing criteria by removing the “Slope” measurement while addressing likelihood difference and taking the correct risk order into consideration while addressing hazard discrimination. Overall, this new evaluation methodology provided more precise evaluation on the staging schemes and had been successfully applied to the development and validation of a staging system for HPV‐related oropharyngeal cancer.^17^

To the best of our knowledge, this is the largest MESCC cohort with longest follow‐up times (over 40 years) to investigate the TNM classifications and staging schemes for MESCC patients by multidimensional criteria. Besides, this study presented the results for each criterion separately, thus allowing the investigators to focus on the characteristics that is most relevant to their specific research purpose. Nevertheless, there are still some limitations as follows. First, some important clinical factors, such as smoking status, alcohol consumption, and comorbidities, were not incorporated into the Cox proportional hazards model due to the restriction of data availability in SEER database. Meanwhile, the sample size was not large enough to investigate the association between treatment type and patients’ prognoses by our staging schemes. Besides, the four N‐classifications (N0–N3) were narrowed to two classifications (N0 and N1–3) due to the insufficient information from SEER database, for instance, lacking the information of size, laterality, and accurate metastasis sites of lymph nodes. Therefore, further studies with larger sample size and more detailed clinical information are needed to investigate the TNM classification systems for MESCC.

In summary, we confirmed that the Stell’s T‐classification was a strong and independent prognostic factor for MESCC patients’ prognosis. Besides, even though the contribution of N‐ and M‐classifications was largely overwhelmed by the T‐classification, their prognostic role could still not be ignored. Meanwhile, our AHR‐Ⅰ staging scheme, a comprehensive staging scheme that integrating T‐, N‐, and M‐classifications, might be an optimal option for clinical practitioners to predict MESCC patients’ prognosis and make proper clinical decisions.

## CONFLICT OF INTEREST

The authors declare that there is no conflict of interest.

## ETHICS STATEMENT

This study was approved by our institutional review board (No. 2019.357), and patient consent was not applicable as SEER data are publicly available.

## Supporting information

Table S1‐S5

## Data Availability

The data that support the findings of this study are available and derived from the following resources available in the public domain: SEER database.
