# Supplementary material for: Evaluating the prognostic contributions of TNM classifications and building novel staging schemes for middle ear squamous cell carcinoma
Source: Cancer Med. 2021 Sep 24;10(22):7958–67. doi: 10.1002/cam4.4306 (PMC8607269; doi:10.1002/cam4.4306)
Supplement: Supplementary file 1 — Table S1‐S5 [file CAM4-10-7958-s001.docx]

**Table S1. Definition of Stell’s T classification and corresponding extent of disease in SEER database**

| Stell’s classification | Definition | SEER extent of disease | Codes for extent of disease in SEER |
| --- | --- | --- | --- |
| T1 | Tumor limited to site of origin i.e., with no facial nerve paralysis and no bone destruction on radiography | In situ: noninvasive OR Invasive tumor confined to site of origin (septum, incus, malleus, stapes, tympanic membrane, cochlea) | 00/10 |
| T2 | Tumor extending beyond the site of origin indicated by facial paralysis or radiologic evidence of bone destruction, but no extension beyond the organ of origin | Localized, NOS OR Adjacent connective tissue (auditory tube, nerve(s)) | 30/40 |
| T3 | Clinical or radiologic evidence of extension to surrounding structures (dura, base of the skull, parotid gland, temporomandibular joint, etc) | Adjacent organs/structures (nasopharynx, mastoid antrum, temporal bone, internal carotid artery, external auditory meatus) OR Further contiguous extension (meninges) | 60/80 |

**Table S2. Univariable Cox model of OS and CSS in MESCC patients**

| **Covariate** | | **OS** | | | **CSS** | | |
| --- | --- | --- | --- | --- | --- | --- | --- |
|  |  | **HR (95%CI)** | **P value** | **Global p-value** | **HR (95%CI)** | **P value** | **Global p-value** |
| Gender | Female | reference |  | ***0.0014**** | reference |  | ***0.0074**** |
|  | Male | 0.60 (0.44, 0.82) | ***0.0014**** |  | 0.56 (0.37, 0.86) | ***0.0074**** |  |
| Marital status | Married | reference |  | 0.27 | reference |  | ***0.017**** |
|  | Unmarried | 1.30 (0.82, 2.06) | 0.27 |  | 1.92 (1.12, 3.28) | ***0.017**** |  |
| Age | $<$60 | reference |  | 0.057 | reference |  | 0.58 |
|  | 60-69 | 1.02 (0.66, 1.58) | 0.92 |  | 1.08 (0.64, 1.83) | 0.78 |  |
|  | ≥70 | 1.48 (1.01, 2.17) | ***0.045**** |  | 0.83 (0.49, 1.4) | 0.49 |  |
| Race | Black | reference |  | 0.22 | reference |  | 0.17 |
|  | Others | 0.51 (0.25,1.03) | 0.061 |  | 0.42 (0.17, 1.00) | 0.051 |  |
|  | White | 0.57 (0.32,1.04) | 0.069 |  | 0.47 (0.23, 0.94) | ***0.033**** |  |
| Treatment | No treatment | reference |  | ***0.046**** | reference |  | ***0.028**** |
|  | RT/CT/CRT | 0.78 (0.46, 1.33) | 0.36 |  | 1.21 (0.56, 2.59) | 0.63 |  |
|  | S only | 0.50 (0.28, 0.9) | ***0.02**** |  | 0.43 (0.17, 1.08) | 0.073 |  |
|  | S+RT/CT/CRT | 0.88 (0.51,1.5) | 0.63 |  | 1.05 (0.48, 2.30) | 0.90 |  |
| Grade | Grade 1 | reference |  | 0.064 | reference |  | 0.44 |
|  | Grade 2 | 0.68 (0.47, 0.99) | 0.12 |  | 0.70 (0.42, 1.18) | 0.19 |  |
|  | Grade 3 | 1.15 (0.74, 1.78) | 0.22 |  | 0.69 (0.35, 1.36) | 0.29 |  |
|  | Grade 4 | 1.07 (0.48, 2.34) | 0.94 |  | 1.41 (0.34, 5.96) | 0.64 |  |
| T classification | T1 | reference |  | ***0.013**** | reference |  | ***0.012**** |
|  | T2 | 1.79 (0.88, 3.64) | 0.11 |  | 5.88 (1.29, 26.91) | ***0.022**** |  |
|  | T3 | 2.37 (1.31, 4.28) | ***0.0041**** |  | 8.11 (1.96, 33.55) | ***0.0039**** |  |
| N classification | N0 | reference |  | 0.67 | reference |  | 0.16 |
|  | N1-3 | 1.13 (0.64,2) | 0.67 |  | 1.64 (0.82,3.28) | 0.16 |  |
| M classification | M0 | reference |  | $<$**0.001*** | reference |  | ***0.0037**** |
|  | M1 | 3.65 (1.75,7.6) | $\boldsymbol{<}$***0.001**** |  | 3.97 (1.57,10.07) | ***0.0037**** |  |

*P$<$0.05, S: surgery; RT: radiotherapy; CT: chemotherapy; CRT: chemoradiation; MESCC: middle ear squamous cell carcinoma; CSS: cause specific survival; OS: overall survival.

**Table S3. 3-year and 5-year overall survival rates and cause specific survival rates of MESCC patients based on various covariates**

| Covariate | Strata | Overall survival (%) | | | | Cause specific survival (%) | | | |
| --- | --- | --- | --- | --- | --- | --- | --- | --- | --- |
|  |  | Events/Total | 3-year (95%CI) | 5-year (95%CI) | p-value | Events/Total | 3-year (95%CI) | 5-year (95%CI) | p-value |
| Age | $<$60 | 44/59 | 40 (29-55) | 29 (19-45) | 0.055 | 37/153 | 79 (73-86) | 76 (69-83) | 0.58 |
|  | 60-69 | 42/59 | 43 (31-58) | 40 (29-56) |  | 39/86 | 57 (46-69) | 57 (46-69) |  |
|  | ≥70 | 78/96 | 37 (28-49) | 26 (18-38) |  | 43/135 | 65 (56-75) | 56 (47-68) |  |
| Treatment | No treatment | 18/24 | 49 (32-74) | 24 (10-57) | ***0.041**** | 8/24 | 71 (54-94) | 46 (23-92) | ***0.02**** |
|  | S only | 35/52 | 54 (42-71) | 51 (39-68) |  | 11/52 | 76 (64-91) | 72 (59-88) |  |
|  | RT/CT/CRT | 55/71 | 36 (26-50) | 27 (18-41) |  | 39/71 | 50 (39-64) | 45 (34-60) |  |
|  | S+RT/CT/CRT | 56/67 | 30 (21-44) | 23 (14-36) |  | 29/67 | 46 (34-62) | 46 (34-62) |  |
| T classification | T1 | 13/23 | 82 (68-100) | 63 (45-90) | ***0.01**** | 2/23 | 95 (85-100) | 87 (71-100) | ***0.0025**** |
|  | T2 | 20/30 | 64 (52-78) | 62 (50-77) |  | 10/30 | 59 (42-84) | 59 (42-84) |  |
|  | T3 | 80/97 | 32 (24-43) | 27 (20-38) |  | 42/97 | 53 (42-65) | 51 (40-64) |  |
| N classification | N0 | 84/113 | 43 (35-54) | 34 (26-45) | 0.68 | 39/113 | 64 (55-75) | 63 (53-74) | 0.16 |
|  | N1-3 | 14/20 | 34 (18-63) | 34 (18-63) |  | 10/20 | 47 (28-79) | 47 (28-79) |  |
| M classification | M0 | 111/146 | 58 (52-65) | 51 (45-58) | $\boldsymbol{<}$***0.001**** | 53/146 | 62 (53-71) | 59 (51-69) | ***0.002**** |
|  | M1 | 8/8 | 0 | 0 |  | 5/8 | 30 (10-94) | 30 (10-94) |  |
| AHR-Ⅰ | stage Ⅰ | 8/18 | 77 (60-100) | 68 (47-97) | ***0.0064**** | 1/18 | 93 (80-100) | 93 (80-100) | ***0.0023**** |
|  | stage Ⅱ | 13/21 | 50 (32-78) | 42 (24-74) |  | 5/21 | 69 (50-97) | 69 (50-97) |  |
|  | stage Ⅲ | 67/83 | 34 (25-47) | 28 (20-41) |  | 49/128 | 54 (43-67) | 51 (40-66) |  |
| AHR-Ⅱ | stage Ⅰ | 8/18 | 77 (60-100) | 68 (47-97) | ***0.0078**** | 1/18 | 93 (80-100) | 93 (80-100) | ***0.0024**** |
|  | stage Ⅱ | 15/24 | 48 (32-74) | 41 (25-70) |  | 6/24 | 68 (50-94) | 68 (50-94) |  |
|  | stage Ⅲ | 65/80 | 34 (25-47) | 28 (20-41) |  | 36/80 | 53 (42-67) | 51 (40-65) |  |
| AHR-Ⅲ | stage Ⅰ | 8/18 | 77 (60-100) | 68 (47-97) | ***0.024**** | 1/18 | 93 (80-100) | 93 (80-100) | ***0.0058**** |
|  | stage Ⅱ | 71/91 | 39 (30-50) | 31 (22-43) |  | 35/91 | 60 (49-72) | 57 (47-71) |  |
|  | stage Ⅲ | 9/13 | 28 (11-70) | 28 (11-70) |  | 7/13 | 38 (17-83) | 38 (17-83) |  |
| ST | stage Ⅰ | 8/18 | 77 (60-100) | 68 (47-97) | ***0.0078**** | 1/18 | 93 (80-100) | 93 (80-100) | ***0.0024**** |
|  | stage Ⅱ | 15/24 | 48 (32-74) | 41 (25-70) |  | 6/24 | 68 (50-94) | 68 (50-94) |  |
|  | stage Ⅲ | 65/80 | 34 (25-47) | 28 (20-41) |  | 36/80 | 53 (42-67) | 51 (40-65) |  |
| Gender | Female | 75/89 | 31 (23-43) | 18 (11-29) | ***0.0013**** | 44/89 | 46 (36-59) | 42 (31-56) | ***0.0069**** |
|  | male | 89/125 | 45 (37-55) | 41 (32-51) |  | 43/125 | 64 (55-74) | 59 (50-70) |  |

*P$<$0.05, S: surgery; RT: radiotherapy; CT: chemotherapy; CRT: chemoradiation; MESCC: middle ear squamous cell carcinoma; CSS: cause specific survival; OS: overall survival.

**Table S4. AHR based on OS and distribution of MESCC patients based on different combinations**

| T-/N- classification | N0 | N1-3 |
| --- | --- | --- |
| T1 | Reference (18) | NA (0) |
| T2 | 1.94 (21) | 2.64 (3) |
| T3 | 3.15 (67) | 4.56 (13) |

Notes: this table shows the number of patients in each combination of T- and N- classification (in the bracket) as well as their corresponding adjusted hazard ratio for overall survival. MESCC: middle ear squamous cell carcinoma.

**Table S5. AHR based on CSS and distribution of MESCC patients based on different combinations**

| AHR (number of participants) | N0 | N1-3 |
| --- | --- | --- |
| T1 | Reference (18) | NA (0) |
| T2 | 5.33 (21) | 11.94 (3) |
| T3 | 10.3 (67) | 12.57 (13) |

Notes: this table shows the number of patients in each combination of T- and N- classification (in the bracket) as well as their corresponding adjusted hazard ratio for overall survival. MESCC: middle ear squamous cell carcinoma.
